# Editorial: Family medicine and primary care: Best practice to achieve health equity for western and traditional Chinese medicine

**DOI:** 10.3389/fmed.2022.1125981

**Published:** 2023-01-10

**Authors:** Xiaomo Xiong, Gang Lv, Xiangxiang Jiang, Hend Mansoor, Kevin Lu

**Affiliations:** ^1^Department of Clinical Pharmacy and Outcomes Sciences, University of South Carolina, Columbia, SC, United States; ^2^Department of General Surgery, The First Medical Center of Chinese PLA General Hospital, Beijing, China; ^3^College of Pharmacy, University of Kentucky, Lexington, KY, United States

**Keywords:** family medicine, primary care, clinical practice, health outcomes, clinical effectiveness

Primary care stresses the importance of the relationship between physicians and patients (1). According to the Institute of Medicine (IOM) Committee, primary care provides integrated, accessible healthcare services by physicians and relevant providers, through developing a sustained partnership with patients and addressing personal healthcare needs in the context of family and community (1, 2). As one of the most significant components of primary care, family medicine is defined as medical specialties that manage common and long-term illnesses, which focus on overall health and well-being (3). Unlike other specialties that are limited to a particular organ or disease, family physicians are the only specialists qualified to treat most ailments and provide comprehensive healthcare for people of all ages (4). In general, family physicians need to know patients' entire medical history when offering primary health care, and they typically receive specialized training in diagnosing (5). Meanwhile, family physicians are usually responsible for offering care to patients of all ages and care to multiple generations at the same time (5).

The studies collected on this topic cover a wide range of research on family medicine and primary care, including systematic review and meta-analysis of the prevalence and risk factors of disease, research exploring factors associated with disease, strategies aimed at improving the healthcare system, effects of different guidelines on mortality risk of disease, development of measurement technology, evaluation the price, availability, and affordability of essential medicines, as well as distribution of health problems. We expect that the research on this topic or the evidence they provide will act an informative role in family medicine and primary care. Community Health Workers (CHWs) are a key component in promoting population health and improving health systems, especially in many low- and middle-income countries (Moh et al.). Many infectious diseases such as malaria, tuberculosis, and human immunodeficiency virus (HIV) are major issues for the health system in Côte d'Ivoire, mainly because health resources remain inaccessible and inefficient, especially in rural areas (Moh et al.). To solve this problem, Moh et al. conducted a study in three locations to improve the integration of HIV, malaria, and tuberculosis prevention. This project was planned for a duration of 3 years, including 2 years of field activities with six main activities (Moh et al.). Through this project, Moh et al. proposed some strategies to improve the integration of HIV, malaria, and tuberculosis prevention, including selecting and strengthening the capacity of community health workers to provide care for these three diseases, providing monthly animation of village health committees by target groups, and using application and tablets for data collection.

This topic also collected a study regarding the policy for patients with hypertension in China, which highlighted how different guidelines would have an impact on the effects of primary care and patients' health outcomes. Specifically, the China Hypertension League (CHL) guideline is different from the guidelines in the United States (6, 7). Therefore, Mai et al. conducted a study, using nationally representative data from the China Health and Retirement Longitudinal Study (CHARLS) with 17,708 subjects aged 45 years and older in 2011–2012, to examine the change in the prevalence and mortality, if the 2017 American College of Cardiology (ACC)/American Heart Association (AHA) hypertension guideline were adopted for Chinese adults. The study found that if the 2017 ACC/AHA guideline was adapted for Chinese adults aged 45 years or older, the hypertension prevalence would increase, which was mostly attributable to the change in the definition of systolic diastolic hypertension (Mai et al.). However, the difference was minuscule in the proportion of people recommended for antihypertensive treatment among people with isolated diastolic hypertension or isolated systolic hypertension. Also, the study has found that the adoption of the 2017 ACC/AHA might be applicable to improve the unacceptable hypertension control rate for Chinese adults aged 45 years or older (Mai et al.).

Another study covered in this Research Topic also focused on primary care for blood pressure in the Chinese population. Previous research suggests that routine blood pressure (BP) monitoring may help predict mortality (8, 9). Therefore, it is beneficial for wearable non-invasive blood pressure measurement (BPM) to help manage BP and relevant health outcomes. Liu Z. et al. conducted a systematic review to explore the status quo of BP measurement technology and development trends of BP measurement. In general, the technology of auscultatory, applanation tonometry, and volume clam methods are mature, and new control methods of volume clamp are developing (Liu Z. et al.). However, the study found limitations and challenges in existing finger-end BPM technologies as well (Liu Z. et al.), indicating that further research on new technologies is still needed to facilitate the development of daily multi-scenario and multi-frequency BPM technologies and provide a basis for realizing long-term personal BP management strategies.

As more and more new medicines become available, healthcare systems face considerable challenges in regulating drug prices, affordability, and affordability for primary care (10). To evaluate the price, availability, and affordability of essential medicines in primary healthcare institutions in Jiangsu Province in China, a mixed longitudinal and cross-sectional survey was conducted by Wang et al. based on the adjusted World Health Organization and Health Action International methodology. This study found that the Medication Possession Ratio (MPR) for lowest-priced generics (LPGs) was generally decreasing between 2016 and 2020 (Wang et al.). In addition, the median availability of generic medicines increased in 2018 (Wang et al.). These results indicated that the National Essential Medicines Policy (NEMP) has proceeded relatively well in the primary healthcare institutions (PHIs) in Jiangsu (Wang et al.). In addition, this Research Topic also contains other related studies, including a systematic review on dyspnea measurement in acute heart failure (Zhang et al.), research exploring factors associated with childhood asthma and wheezing in Chinese preschool-aged children (Deng et al.), a systematic review and meta-analysis of the prevalence and risk factors of depression in type 2 diabetes patients in China (Liu X. et al.), and a cross-sectional study exploring the distribution of health problems at the general outpatients' clinic of the University of Hong Kong-Shenzhen Hospital (Chen et al.). It has long been documented that characteristics such as gender, race, and socioeconomic status are associated with the use of healthcare services in routine care, which might further bring health disparities in health outcomes (11–13). As the role of family physicians is to interact with patients from all groups, family medicine could provide the capability of collaborating with national stakeholders to reduce health disparities in the United States (14, 15). Furthermore, the evidence on health disparities could act as a guide for family physicians as guidance in clinical practice to notice the disparities, thereby further achieving health equity for patients with different characteristics and socioeconomic statuses (15–17). At last, we expect more studies and research to be conducted to support the value-based healthcare scheme and to achieve health equity.

## Author contributions

XX, GL, XJ, HM, and KL conceived the idea for the editorial and wrote the initial draft. All authors approved the final version of the editorial.
